# High-grade salivary gland cancer: is surgery followed by radiotherapy an adequate treatment to reach tumor control? Results from a tertiary referral centre focussing on incidence and management of distant metastases

**DOI:** 10.1007/s00405-021-07024-9

**Published:** 2021-08-26

**Authors:** Viola Freitag, Sebastian Lettmaier, Sabine Semrau, Markus Hecht, Konstantinos Mantsopoulos, Sarina K. Müller, Maximillian Traxdorf, Heinrich Iro, Abbas Agaimy, Rainer Fietkau, Marlen Haderlein

**Affiliations:** 1 Department of Radiation Oncology, University Hospital of Erlangen, Friedrich -Alexander-University Erlangen-Nürnberg (FAU), Universitätsstraße 27, 91054 Erlangen, Germany; 2 Department of Otorhinolaryngology, Head and Neck Surgery, University Hospital of Erlangen, Friedrich-AlexanderUniversity Erlangen-Nürnberg (FAU), Erlangen, Germany; 3 Institute of Pathology, University Hospital of Erlangen, Friedrich-Alexander-University Erlangen-Nürnberg (FAU), Erlangen, Germany

**Keywords:** Salivary gland cancer, Distant metastases, High-grade, Radiotherapy, Chemotherapy

## Abstract

**Purpose:**

Salivary Gland cancer (SGC) is a rare and heterogenous group of tumors. Standard therapeutic options achieve high local but poor distant control rates, especially in high-grade SGC. The aim of this monocentric study was to evaluate patterns of recurrence and its treatment options (local ablative vs. systemic) in a homogenously treated patient population with high-grade SGC after surgery and radio(chemo)therapy.

**Methods:**

Monocentric, retrospective study of patients with newly diagnosed high-grade salivary gland cancer. We retrospectively reviewed clinical reports from 69 patients with high-grade salivary gland cancer in a single-center audit. Survival rates were calculated using the Kaplan–Meier method and prognostic variables were analyzed (univariate analysis: log-rank test; multivariate analysis: Cox regression analysis).

**Results:**

The median time of follow-up was 31 months. After 5 years, the cumulative overall survival was 65.2%, cumulative incidence of local recurrence was 7.2%, whereas the cumulative incidence of distant metastases was 43.5% after 5 years. 30 of 69 patients developed distant metastases during the time of follow-up, especially patients with adenoid cystic carcinoma, salivary duct carcinoma, adenocarcinoma NOS and acinic cell carcinoma with high-grade transformation. The most common type of therapy therefore was chemotherapy (50%). 85.7% of patients with local ablative therapy of distant metastases show disease progression during follow-up afterwards.

**Conclusion:**

With surgery and radio-chemotherapy, a high rate of loco-regional control is reached, but over 40% of patients develop distant metastases in the further follow-up which usually present a diffuse pattern involving in a diffuse metastases. Therefore, in the future, intensified interdisciplinary combination therapies even in the first-line treatment in certain subtypes of high-grade SGC should be investigated.

**Supplementary Information:**

The online version contains supplementary material available at 10.1007/s00405-021-07024-9.

## Introduction

Salivary gland cancer (SGC) is a rare disease (0.6–1.4 per 100,000 [1]) including various histological tumor subtypes (more than 20 according to the WHO classification of head and neck cancers of 2017) [2]. The established standard therapy is surgery and, in case of locally advanced disease, postoperative radio(chemo)therapy [3–7]. In this way, local control rates of about 90% are achieved, but a large percentage of patients develop distant metastases resulting in a decrease in overall and disease-specific survival [8].

In high-grade salivary gland cancer, up to 50% of patients show distant metastases on further follow-up [9, 10]. Standard chemotherapy schedules in these patients provide low response rates, even using triplet combinations [11].

Some studies suggest that patients with metastatic SGC, especially patients with adenoid cystic carcinomas (AdCC), benefit from local therapeutic options [12, 13].

In this retrospective study, a homogenously treated patient population with high-grade SGC who had undergone surgery and postoperative radio(chemo)therapy is evaluated regarding incidence and location of distant metastases and their respective treatment options (local ablative vs. systemic).

## Design

### Data collection, patient characteristics

The basis for data collection was provided by the clinical reports from 69 patients with previously untreated high-grade salivary gland cancer who had previously undergone surgery followed by photon radio(chemo)therapy from December 1999 to May 2020 at the Department of Radiation Oncology in Erlangen.

We excluded patients with low- or intermediate-grade SGC, squamous cell carcinomas and distant metastases at the time of first diagnosis.

All tumors were classified according to the TNM classification system of 2017.

Detailed information about patient characteristics is shown in Table 1.

**Table 1:** Patient characteristics

Patient characteristics (*n* = 69)		
Characteristics	No. of patients	%
Sex		
Female	26	37.7
Male	43	62.3
Age at diagnosis [y]		
Median	58	
Range	26–83	
Primary tumor site		
Parotid gland	48	69.8
Submandibular gland	8	11.6
Minor salivary gland	7	10.1
Nose and paranasal sinuses	5	7.2
Acoustic duct	1	1.4
Histologic subtypes		
Salivary duct carcinoma	21	30.4
Adenoid cystic carcinoma (G3)	16	23.2
Muco-epidermoid carcinoma (G3)	14	20.3
Adenocarcinoma NOS	9	13
Acinic cell carcinoma with high-grade transformation (G3)	3	4.3
Other (oncocytic, myoepithelial and secretory carcinoma)	6	8.6
T classification at first diagnosis		
T1	6	8.7
T2	13	18.8
T3	20	29
T4	30	43.5
N classification at first diagnosis		
N0	22	31.9
N1	16	23.2
N2b	27	39.1
N3	4	5.8
Resected lymph nodes		
Median	21	
Range	0–58	
Positive lymph nodes		
Median	1	
Range	0–30	
Perinodal spread at first diagnosis		
No	37	53.6
Yes	26	37.7
Unknown	6	8.7
Lymphangiosis at first diagnosis		
L0	48	69.6
L1	19	27.5
Lx	2	2.9
Hemangiosis at first diagnosis		
V0	57	82.6
V1	10	14.5
Vx	2	2.9
Perineural spread at first diagnosis		
Pn0	17	24.6
Pn1	46	66.7
Pnx	5	7.2
Resection margins		
R0	52	75.4
R1	11	15.9
R2	2	2.9
Rx	4	5.8
Neck dissection		
None	4	5.8
Ipsilateral	56	81.2
Bilateral	9	13
Radio-chemotherapy		
Radiation alone	17	24.6
Radio-chemotherapy	52	75.4
Planning target volume		
Primary tumor region	11	15.9
Primary tumor region, ipsilateral neck	39	56.5
Primary tumor region, bilateral neck	19	27.5
Applicated dose of radiotherapy [Gy]		
Median	64	
Range	45.0–74.0	
Technique of radiotherapy		
3D	37	53.6
IMRT	6	8.7
VMAT	26	37.7
Tobacco consumption		
Never	45	65.2
Yes	6	8.7
Quit earlier	15	21.7
Unknown	3	4.3

### Treatment

All patients were treated with surgery followed by photon radiotherapy or photon radio-chemotherapy in curative intention.

Local resection of the primary tumor was performed in all patients, mostly combined with neck dissection in 65 patients (94.2%). 56 patients (81.2%) underwent unilateral neck dissection and 9 patients (13.0%) underwent bilateral neck dissection. Bilateral neck dissection was performed if there was evidence for contralateral neck lymph node metastasis on ultrasound or if the primary tumor was located at or crossing the midline (e.g. in cases of primary tumors arising from submandibular or minor salivary glands). Only four patients (5.8%) had surgery without neck dissection.

We defined the date of biopsy or (first) surgery (in case there was no preoperative biopsy) to the primary tumor as the date of first diagnosis.

The indications for postoperative radiation in SGC are based on retrospective trials [3, 4, 14]. In detail, these indications include locally advanced tumor stage (classified as pT3/pT4), bone invasion, peri-nodal or peri-neural spread, close or positive resection margins, high-grade histology and all adenoid cystic carcinomas except for tumors classified as pT1. As all of our patients had high-grade carcinomas, every patient underwent adjuvant radiotherapy.

The median radiotherapy dose was 64 Gy (range 45.0–74.0 Gy). Radiotherapy usually was applied up to 64 Gy in high-risk region (primary tumor region, peri-nodal spread) and in case of suspected macroscopic tumor after resection a dose of a minimum of 70 Gy was delivered. Single fraction dose was 2 Gy. In one patient, a hypo-fractionated accelerated radiotherapy up to 45 Gy with a single dose of 3 Gy (EQD 2 value (α/ß = 10): 48.75 Gy) was performed because of advanced age and reduced ECOG performance status of the patient.

3D-conformal radiotherapy was performed in 37 patients (53.6%), whereas 34 patients (46.4%) underwent volumetric-modulated arc therapy or intensity-modulated radiotherapy. The clinical target volume always included the region of the primary tumor and the nerve tracts up to the base of the skull. If there were positive ipsilateral neck nodes, this region was also included in the clinical target volume (39 patients, 56.5%). In case of multiple positive ipsilateral neck, nodes with extracapsular spread or primary tumor at/crossing the midline, bilateral neck nodes were additionally included (19 patients, 27.5%). Patient positioning was checked daily with portal imaging or cone-beam-ct.

Concomitant platinum-based chemotherapy was usually recommended in locally advanced situations (pT3/pT4), peri-nodal spread, ≥ three lymph node metastases or positive/close resection margins. Patients with any of these tumor characteristics would have been treated with chemotherapy unless they either refused or had comorbidities that did not allow chemotherapy. 52 of our patients (75.4%) received combined postoperative radio-chemotherapy.

After radio(chemo)therapy, we planned follow-up visits for every patient at regular intervals at the Department of Otorhinolaryngology, Head and Neck Surgery as well as at our own Department of Radiation Oncology in Erlangen. Usually, follow-up visits are scheduled every 3 months for the first 2 years after treatment, then every 6 months from year 3 to 5 after treatment and as yearly follow-up visits afterwards. During follow-up visits, patients underwent an examination of the ear, nose and throat and an ultrasound scan of the parotid region and the neck (Levels I–V). During the first 5 years after treatment, patients had a CT of their chest (usually including the upper abdomen) every 6 months. If patients developed symptoms pointing towards distant metastases, they underwent additional investigations according to their symptoms (mostly additional diagnostic imaging and, if appropriate, tissue sampling).

In case of occurrence of distant metastases, local therapeutic options were evaluated in every patient.

### Main outcome measures

The statistical analysis was carried out using IBM SPSS version 26 (IBM Corporation, Armonk, NY, USA).

Overall survival (OS), disease-free survival (DFS), local-recurrence-free survival (LRFS) and distant-metastases-free survival (DMFS) were calculated using the Kaplan–Meier method from the date of first diagnosis till the date of a patient’s death/disease recurrence/loco-regional recurrence/occurrence of distant metastases or the last available follow-up visit. To correlate patient-related, tumor-related and treatment-related factors with OS, DFS, LRFS and DMFS in a univariate analysis, the log-rank test was used. Considering the limited number of included patients, the following dichotome risk-classification was used: gender, age (< 70 vs. ≥ 70 years), tobacco consumption (never vs. yes/earlier), hypertension (no vs. yes), primary tumor site (parotid vs. non-parotid), histologic subtype (adenoid cystic vs. non-adenoid cystic), T classification (T1/2 vs. T3/4), primary tumor size (≤ 3 cm vs. > 3 cm), N classification (N0 vs. N1/2), number of metastatic lymph nodes (≤ 1 vs. > 1), perinodal spread, lympho-vascular and vascular invasion, perineural spread, neck dissection (no vs. yes), number of dissected lymph nodes (< 10 vs. ≥ 10 and ≤ 27 vs. > 27), lymph node density (≤ 4% vs. > 4%), resection margins (R0 vs. R1/X), second primary tumor resection, planning target volume (primary tumor region vs. primary tumor region and regional lymph node drainage), radiation dose (< 64 Gy vs. ≥ 64 Gy), radiation technique (3D vs. IMRT/VMAT) and postoperative treatment (radiation vs. radio-chemotherapy).

Only variables with a *ρ* value of ≤ 0.05 were included in multivariate analysis using Cox regression. Two-sided *ρ* values of < 0.05 were considered statistically significant.

## Results

The median time of follow-up was 31 months (range 2–182 months).

The median time interval from first diagnosis to the beginning of radio(chemo)therapy was 56 days (range 29–131) and 100 days (range 51–217) from first diagnosis to the end of radio(chemo)therapy.

Most of the patients showed a locally advanced tumor stage at first diagnosis. 73% of the patients had a locally advanced T-stage (pT3 or pT4), 68% of the patients had lymph node metastases and 66% had perineural spread.

### Overall survival (OS)

The estimated cumulative OS was 91.3% after one year, 76.8% after two and 65.2% after five years.

### Disease-free survival (DFS)

**Fig. 1:**
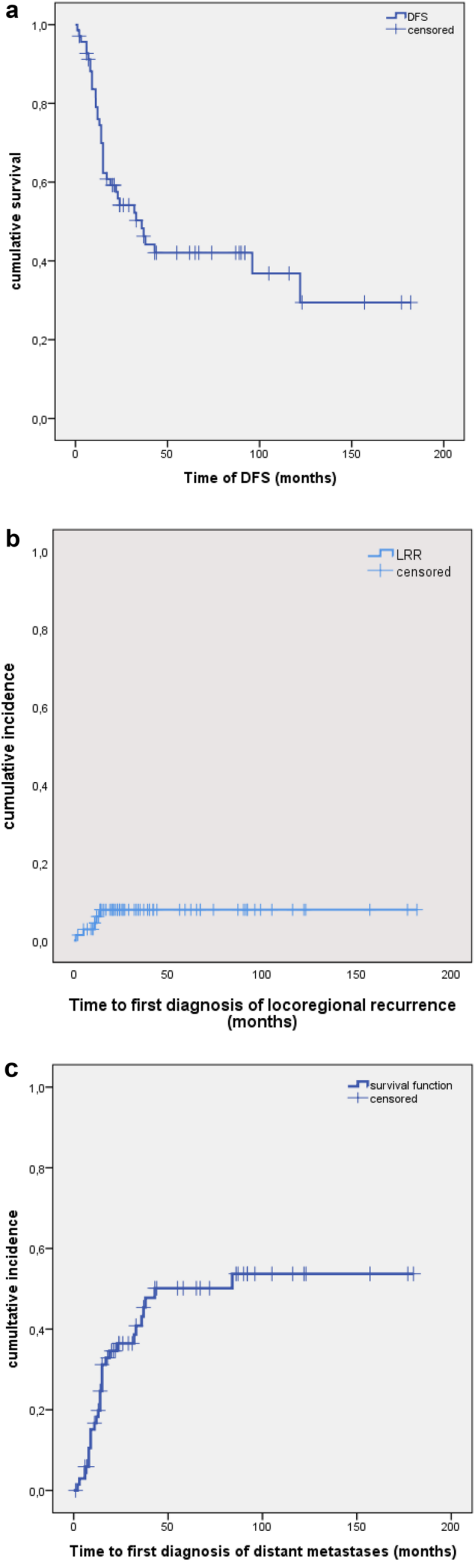
**a** Time of disease-free survival (DFS) in months. **b** Time to first diagnosis of loco-regional recurrence (LRR) in months. **c** Time to first diagnosis of distant metastases in months

**Fig. 2:**
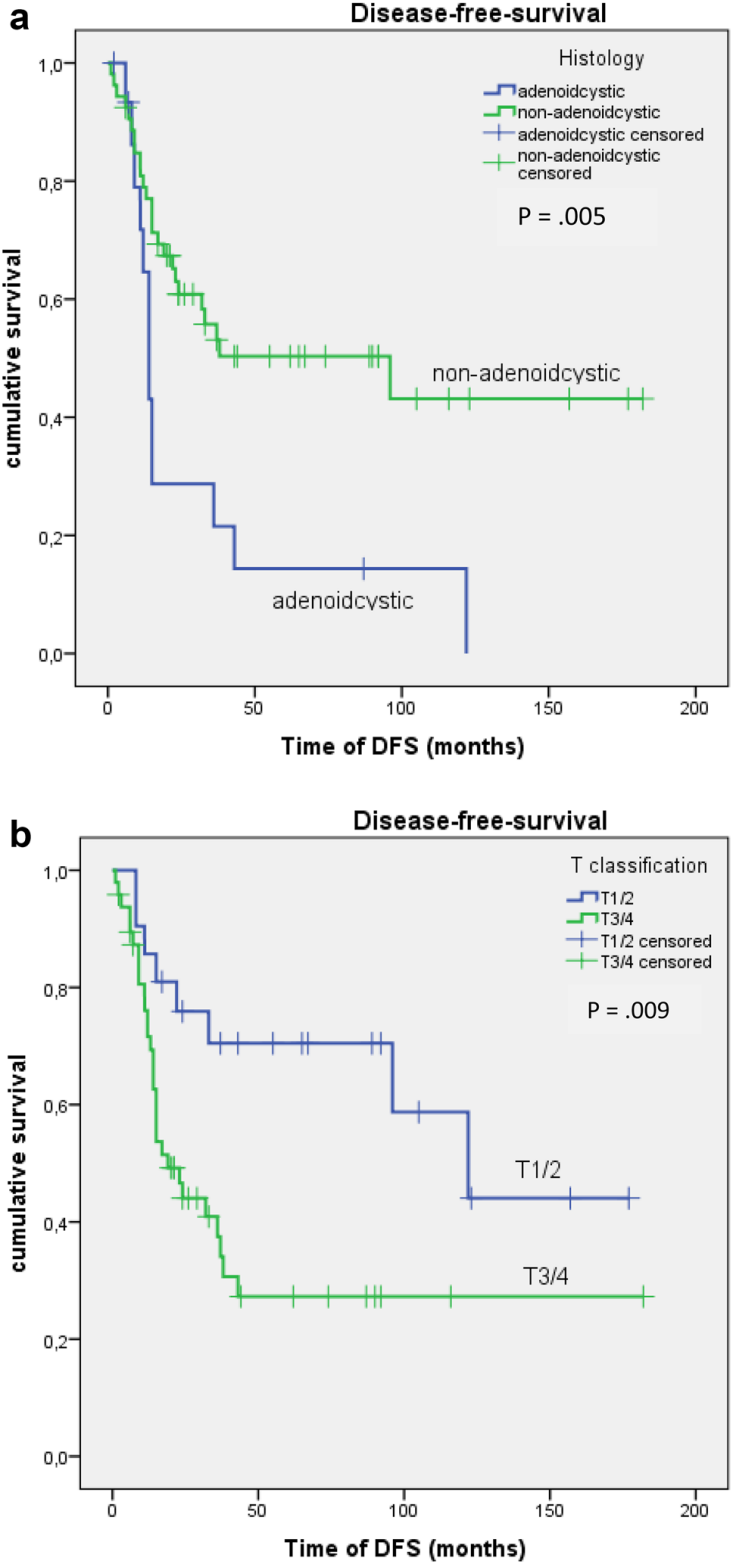
**a** Disease-free survival depending on the histologic subtype. **b** Disease-free survival depending on the T classification

The estimated cumulative DFS was as follows: 76.8% after one year, 56.5% after two years and 47.8% after five years (see Fig. 1a). On univariate analysis, a significant association was shown between DFS and the histologic subtype (*ρ* = 0.005; see Fig. 2a) as well as the T classification (*ρ* = 0.009; see Fig. 2b). Both of these tumor-related factors were also significant on multivariate analysis using Cox regression (see Table S2 and S3 in the supplementary information). A higher T classification and an adenoid cystic histologic subtype were associated with a significantly lower DFS.

### Local-recurrence-free survival (LRFS)

The estimated cumulative LRFS was 94.2% after one year and 92.8% after two and five years (see Fig. 1b).

The association between LRFS and the performance of a neck dissection showed a significant result (*ρ* = 0.044) on univariate analysis. Patients with no neck dissection had a significantly lower LRFS.

### Distant-metastases-free survival (DMFS)

The estimated cumulative DMFS was 79.7%, 65.2% and 56.5% after one, two and five years (see Fig. 1c).

**Fig. 3:**
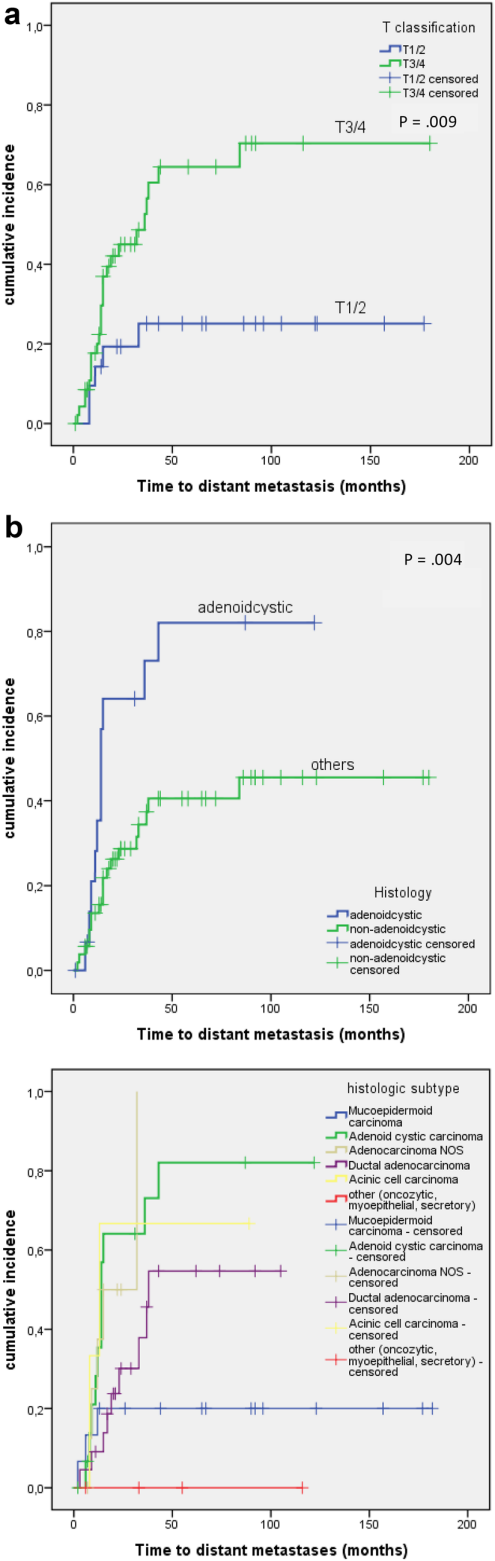
**a** Incidence of distant metastases with respect to T classification. **b** Incidence of distant metastases with respect to the histologic subtype

On univariate analysis, a significantly lower DMFS was associated with a higher T classification (*ρ* = 0.009) and an adenoid cystic histology (*ρ* = 0.004). See also Fig. 3a, b for reference. In multivariate analysis, both factors remained significant (see Table S2 and S3 in the supplementary information).

### Management of distant metastases (DM)

**Fig.4:**
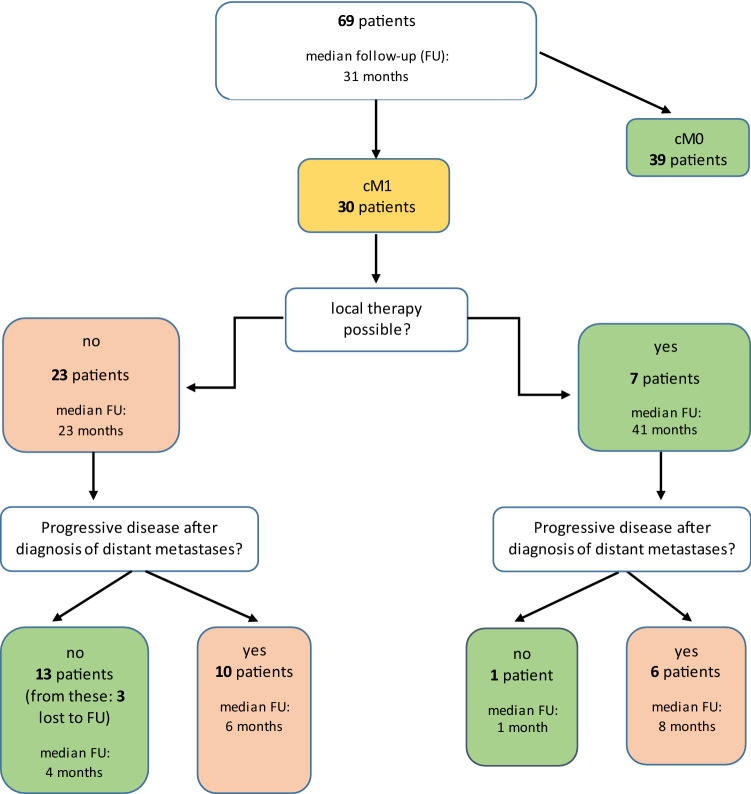
Breakdown of patients with distant metastases

30 (43.5%) of our 69 included patients developed distant metastases during their follow-up. The median time from the date of first diagnosis to the time of DM diagnosis was 13.5 months (range 2–43). The median time of follow-up after the diagnosis of distant metastases was 12 months (range 0–80). For detailed information, see Fig. 4.

The most common anatomical site for the appearance of distant metastases was the lung (73.3%), followed by bone (20%) and the brain and non-regional lymph nodes each accounting for 10% of the total. Other localizations included the liver, the abdominal wall and the pleura. Seven patients (23.3%) developed distant metastases at multiple anatomical sites (defined as two or more involved organ systems), whereas 23 patients (76.7%) developed their distant metastases at one single anatomical site.

In seven out of 30 patients (23.3%), local ablative therapy to distant metastasis was possible. These patients underwent local ablative therapy for oligo-metastatic disease only without systemic therapy. In patients with local ablative therapeutic options, six patients underwent surgery (five resection of lung metastases, one resection of a peritoneal metastases) and one patient underwent heavy ion radiotherapy of a sella metastases. Six of these seven patients (85.7%) showed disease progression after a median time of 10 months (range 2–23) following local therapy to distant metastases. Disease progression after local therapy was disseminated in multiple organs in 3 patients, in the same organ with further local treatment options in 2 patients and in-Radiation-Field in one patient.

Among patients with no local ablative therapeutic options of distant metastases patients underwent the following treatments: 15 patients palliative chemotherapy, two patients palliative chemotherapy and radiotherapy of brain metastases (used agents were Cisplatin/Carboplatin combined with Docetaxel and Trastuzumab), two palliative radiotherapy of bone metastases, two best supportive care and two were lost to follow-up after diagnosis of DM. Chemotherapy was mostly platinum-based (eleven out of 15 patients, 73.3%) using combinations with taxanes (five patients, 33.3%) and/or monoclonal antibodies (two patients, 13.3%). The exactly used combinations were as followed: Cisplatin and Docetaxel (two patients, 13.3%), Cisplatin and Paclitaxel (one patient, 6.7%), Cisplatin and Cetuximab (6.7%), Cisplatin and Vinorelbine (6.7%), Carboplatin mono (13.3%), Carboplatin and Etoposid (6.7%), Carboplatin and Paclitaxel (6.7%), Carboplatin and Pemetrexed (6.7%), Carboplatin combined with Docetaxel and Trastuzumab (6.7%) and Vinorelbine mono (13.3%). In two cases (13.3%), the administered chemotherapy was unknown.

Another sixteen patients (53.3%) showed disease progression after diagnosis and treatment of distant metastasis. The median time from first diagnosis of distant metastases to diagnosis of renewed disease progression was 9 months (range 2–23). The median time of follow-up after progressive disease was 7 months (range 2–51). Out of these sixteen patients, the treatment of 10 patients with furthermore progressive disease was reproducible. 50% received a palliative chemotherapy. The administered combinations were as follows: Cisplatin and Vinorelbine, Trastuzumab and Lapatinib, Trastuzumab and Paclitaxel and Carboplatin mono. In one case, the used drugs were unknown. The other patients received chemotherapy (Carboplatin and Paclitaxel) combined with whole-brain radiotherapy (WBRT), palliative radiotherapy of bone metastases and palliative WBRT (one patient each, 10%). In case of oligo-progressive disease, one patient underwent surgery and one patient surgery and radiotherapy.

**Fig. 5:**
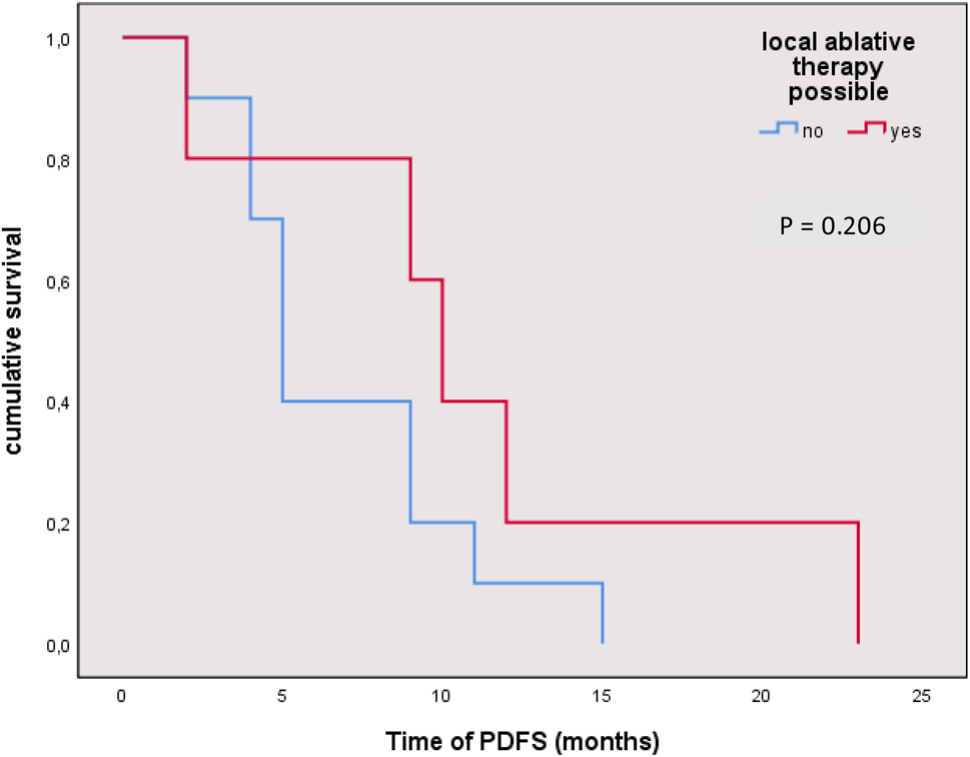
Time of progressive-disease-free survival (PDFS) after first diagnosis of distant metastases

Comparing patients who underwent local ablative therapy after first diagnosis of distant metastases to those who were not eligible for local ablative therapy, there is no significant survival benefit (*p*-value: 0.206). See Fig. 5.

## Discussion

In this study, we analysed data from a rather homogeneously treated group of patients suffering from newly diagnosed high-grade salivary gland cancer who had been treated with surgery and adjuvant radio(chemo)therapy at a single institution to evaluate local and distant control rates. The special focus of this study has been on the incidence of distant metastases as well as their respective treatment (focussing on local ablative vs systemic treatment) options because this is known to be the first site of disease recurrence.

To the best of our knowledge, other existing retrospective studies report data either from heterogeneously treated patients populations or populations with heterogeneous tumor grading, making this the only study which includes high-grade SGC patients treated with surgery followed by radio(chemo)therapy only.

Our results for overall survival were 91.3% after one year, 76.8% after two years and 65.2% after five years and for disease-free survival of 76.8, 56.5 and 47.8% after one, two and five years, respectively. Higher rates of OS and DFS were shown in studies with a patient population inhomogeneous with regard to the tumor grade [15, 16]. This might be the reason for our lower rates of OS and DFS in a cohort of patients with high-grade histology only in keeping with retrospective studies like that by Nam et al.[8]. They showed significantly lower recurrence-free survival in patients with high-grade histology. A previous clinical trial from our study group has shown that, comparing low- and intermediate-grade SGC to high-grade SGC, high-grade histology is the only significant predictor for a decreased DFS and DMFS independent of the tumor subtype [9].

Investigating only locally advanced high-grade tumours after postoperative radio(chemo)therapy on multivariate analysis, we found that patients with adenoid cystic carcinoma (AdCC) of the salivary glands had a significant lower DFS and a higher incidence of distant metastases. Within our cohort of high-grade SGC, the group of patients with adenoid cystic carcinoma had the shortest time from the date of first diagnosis to the date of first diagnosis of distant metastases when compared with other histologic subtypes. This is verified by the results of Mimica et al. [17]. They demonstrated high rates of local and distant metastases and subsequently a lower rate of DFS in patients with AdCC. Amit et al. [18] for example figured a DFS of 68% after 5 years in patients with AdCC of the head and neck irrespective of the histologic grade of the tumor. It has to be mentioned that in this statistical analysis adenoid cystic histology was compared to non-adenoid cystic histology. Comparing all histologic subtypes, patients with adenocarcinoma NOS, salivary duct carcinoma and acinic cell carcinoma with a high-grade transformation also develop distant metastases soon after first diagnosis, whereas muco-epidermoid carcinoma and other rare histologies with high-grade transformations have a lower risk of developing distant metastases.

Other significant associations in our study were found between locally advanced tumours staged as T3 or T4 and a lower DFS and higher incidence of distant metastases. These results were also confirmed by Nam et al.[8] in an analysis including all kinds of tumor grades.

Surgery followed by radiotherapy as local treatment options in high-grade tumours reached a high local control rate even in our population with high-grade SGC only. The cumulative incidence of local recurrence was 5.8% after one year and 7.2% after two and five years, respectively. This is comparable to local control rates reported by other clinical trials [4, 15, 16]. Our study showed a significantly lower rate of local control in patients who did not undergo neck dissection. This result might, however, be interpreted with caution as only four out of our patients (5.8%) had no neck dissection.

In contrast to the high rate of LRFS, the rates of DMFS and DFS are distinctly lower. Our results showing a cumulative incidence of distant metastases of 20.3% after one year, 34.8% after two years and 43.5% after five years and were quite similar to those previously published by other authors [9, 19]. Only in cohorts including low- and intermediate-grade tumors, was the DMFS notably higher [8, 15, 19].

As the development of distant metastases is the main type of treatment failure in SGC, many previous studies recommend local therapeutic options to improve overall outcome [1, 13], and to improve the outcome of adenoid cystic carcinomas in particular [4, 12]. Our results lead us to hypothesize that this fact is not transferable to high-grade situations in general. 43.5% of our patients developed distant metastases (23.3% of the patients developed cM1 at multiple anatomical sites) during their follow-up. Although evaluating local treatment options in all patients, in only 23.3% of the patients with cM1 a local ablative therapy of the distant metastases was possible. The other patients showed diffuse metastatic disease. 85.7% of the patients who underwent local ablative therapy showed progression afterwards. One patient (14.3%) showed no progression after local ablative therapy of distant metastases but was lost to follow-up already 1.5 months after first diagnosis of distant metastases. There was no significant survival benefit in patients undergoing local ablative therapeutic options compared to those who were not. Survival curves show a slightly survival advantage that might be statistically significant in a high number of patients. But it should also be considered that in the group of patients without local ablative therapy there were patients with diffuse metastatic disease/higher tumor burden and patients who underwent systemic therapy or best supportive care only.

Most of the DM rates reported in previously published articles are much lower with values of around 20% [8, 15]. This may be caused by including patients with all kinds of tumor grading and all kinds of tumor stages. In our study, only patients with high-grade locally advanced tumours were included.

Another therapeutic option is the administration of systemically effective cytostatic drugs, but the effect of the standard, mostly platinum-based, regimes is highly limited [17, 20, 21]. Over the last few years, several different target-relevant mutations of key oncogenes and receptor expressions have been detected in the different subtypes of SGC, especially in salivary duct carcinomas (SDC) and in mammary analog secretory carcinomas [22], 23 but last ones not being classified as high-grade tumors. In SDC, Her2-amplification, expression of androgen receptors and other aberrations like RET-fusion are relevant therapeutic targets [23]. Trastuzumab-based systemic therapy is still not approved for the treatment of SGC up to now. In the palliative setting, phase II-trials showed promising response and OS rates in patients with SDC undergoing trastuzumab-based therapy [24] or androgen deprivation [25, 26]. Some retrospective case series also demonstrated improved DFS and OS in patients receiving those therapies in the postoperative setting [27, 28].

C-kit expression is common in most cases of AdCC, but phase II studies showed no effect of Imatinib in metastatic AdCC so far [29]. Effective treatment options suggested by phase II-studies include the EGFR-inhibitor Cetuximab [30] and the multi-tyrosine kinase-inhibitor Lenvatinib [31]. First studies on mono immunotherapy in PD-L1-positive patients with SGC (CPS > 1%) show low objective response rates of 12% [32].

In the future, modern systemic therapy options should be evaluated and perhaps integrated in first-line therapy schedules for patients with different subtypes of high-grade SGC like ACC, salivary duct carcinoma, AciCC with high-grade transformation and adenocarcinoma NOS to prevent distant metastases and increase DFS. Local ablative therapies alone are unlikely to be successful in metastatic high-grade SGC, but modern multi-modal and inter-disciplinary treatment strategies may lead to long-term tumor control.

Moreover in the future, radiologic and biologic markers like known from squamous cell carcinoma may help to select patients for intensified first-line treatment strategies [33–36].

Discussing our results, we also have to pay attention to the limitations of this study. As a result of the small number of included patients, the retrospective design of the study and the short median time of follow-up, the evaluation of treatment outcomes is difficult. Furthermore, we recorded a certain number of patients who failed to attend their prescheduled follow-up visits, including a substantial number of four patients (13.3% of the patients with distant metastases, 5.8% of all patients) with distant metastases who statistically were most unlikely to still be alive. This fact may impact the survival rates. A further limitation of the study is the fact that patients who were not eligible for local therapy often decided to undergo systemic therapy in hospitals near their hometowns, and therefore systemic therapy regimes are diverse and not comparable to each other and often information on further treatment is missing.

Nevertheless, this monocentric study is the first of its kind evaluating the outcome of patients with high-grade SGC only after homogenously prescribed radio(chemo)therapy. It shows that high-grade SGC develops fast after first-line treatment distant metastases that usually may not be treated by local ablative therapeutic options only. Therefore, the study demonstrates the aggressiveness of high-grade SGC and the need for integrating modern systemic therapies in the first-line treatment to decrease the incidence of distant metastases.

## Conclusion

While a high rate of LRFS is observed in patients with high-grade SGC, the prognosis is largely determined by the high rate of distant metastases. These often occur at multiple anatomical sites, so that local ablative therapy options are quite limited. Furthermore, we recorded many cases of disease progression following the diagnosis of distant metastases, even when those had previously been treated with local ablative therapy. The goal for the future, therefore, has to be the development of more effective therapeutic options especially for patients with distant metastases and even better the integration of modern systemic therapy in a multimodal interdisciplinary first-line treatment to prevent distant metastases.

## Supplementary Information

Below is the link to the electronic supplementary material.

Supplementary file1 Univariate analysis of patient-, tumor- and treatment-related parameterst o DFS and DMFS (DOCX 17 KB)

Supplementary file2 Multivariate analysis (DOCX 12 KB)

## References

[1] Jang JY (2018). Treatment outcomes in metastatic and localized high-grade salivary gland cancer: high chance of cure with surgery and post-operative radiation in T1–2 N0 high-grade salivary gland cancer. BMC Cancer.

[2] Ihrler S (2018). Updates on tumours of the salivary glands: 2017 WHO classification. Pathologe.

[3] Terhaard CH (2005). The role of radiotherapy in the treatment of malignant salivary gland tumors. Int J Radiat Oncol Biol Phys.

[4] Garden AS (1995). The influence of positive margins and nerve invasion in adenoid cystic carcinoma of the head and neck treated with surgery and radiation. Int J Radiat Oncol Biol Phys.

[5] von der Grun J (2021). Patterns of care, toxicity and outcome in the treatment of salivary gland carcinomas: long-term experience from a tertiary cancer center. Eur Arch Otorhinolaryngol.

[6] Ruhle A (2020). Radiation-induced toxicities and outcomes after radiotherapy are independent of patient age in elderly salivary gland cancer patients: results from a matched-pair analysis of a rare disease. Eur Arch Otorhinolaryngol.

[7] Geiger JL (2021). Management of salivary gland malignancy: ASCO guideline. J Clin Oncol.

[8] Nam SJ (2016). Risk factors and survival associated with distant metastasis in patients with carcinoma of the salivary gland. Ann Surg Oncol.

[9] Haderlein M (2016). High-grade histology as predictor of early distant metastases and decreased disease-free survival in salivary gland cancer irrespective of tumor subtype. Head Neck.

[10] Westergaard-Nielsen M (2021). Salivary gland carcinoma in Denmark: a national update and follow-up on incidence, histology, and outcome. Eur Arch Otorhinolaryngol.

[11] Wang X (2017). Management of salivary gland carcinomas - a review. Oncotarget.

[12] Chen AM (2006). Adenoid cystic carcinoma of the head and neck treated by surgery with or without postoperative radiation therapy: prognostic features of recurrence. Int J Radiat Oncol Biol Phys.

[13] Zeidan YH (2015). Survival benefit for adjuvant radiation therapy in minor salivary gland cancers. Oral Oncol.

[14] Armstrong JG (1990). Malignant tumors of major salivary gland origin. A matched-pair analysis of the role of combined surgery and postoperative radiotherapy. Arch Otolaryngol Head Neck Surg.

[15] Hosni A (2016). Outcomes and prognostic factors for major salivary gland carcinoma following postoperative radiotherapy. Oral Oncol.

[16] Terhaard CH (2004). Salivary gland carcinoma: independent prognostic factors for locoregional control, distant metastases, and overall survival: results of the Dutch head and neck oncology cooperative group. Head Neck.

[17] Mimica X (2020). Distant metastasis of salivary gland cancer: incidence, management, and outcomes. Cancer.

[18] Amit M (2014). Analysis of failure in patients with adenoid cystic carcinoma of the head and neck. Intern Collab Study Head Neck.

[19] Ali S (2015). Distant metastases in patients with carcinoma of the major salivary glands. Ann Surg Oncol.

[20] Amini A (2016). Association of adjuvant chemoradiotherapy vs radiotherapy alone with survival in patients with resected major salivary gland carcinoma: data from the national cancer data base. JAMA Otolaryngol Head Neck Surg.

[21] Mifsud MJ (2016). Adjuvant radiotherapy versus concurrent chemoradiotherapy for the management of high-risk salivary gland carcinomas. Head Neck.

[22] Cros J (2013). Expression and mutational status of treatment-relevant targets and key oncogenes in 123 malignant salivary gland tumours. Ann Oncol.

[23] Wang K (2016). Profiling of 149 salivary duct carcinomas, carcinoma ex pleomorphic adenomas, and adenocarcinomas, not otherwise specified reveals actionable genomic alterations. Clin Cancer Res.

[24] Takahashi H (2019). Phase II trial of trastuzumab and docetaxel in patients with human epidermal growth factor receptor 2-positive salivary duct carcinoma. J Clin Oncol.

[25] Fushimi C (2018). A prospective phase II study of combined androgen blockade in patients with androgen receptor-positive metastatic or locally advanced unresectable salivary gland carcinoma. Ann Oncol.

[26] Locati LD (2016). Clinical activity of androgen deprivation therapy in patients with metastatic/relapsed androgen receptor-positive salivary gland cancers. Head Neck.

[27] Hanna GJ (2020). The benefits of adjuvant trastuzumab for HER-2-positive salivary gland cancers. Oncologist.

[28] van Boxtel W (2019). Adjuvant androgen deprivation therapy for poor-risk, androgen receptor-positive salivary duct carcinoma. Eur J Cancer.

[29] Pfeffer MR (2007). A phase II study of Imatinib for advanced adenoid cystic carcinoma of head and neck salivary glands. Oral Oncol.

[30] Locati LD (2009). Cetuximab in recurrent and/or metastatic salivary gland carcinomas: a phase II study. Oral Oncol.

[31] Tchekmedyian V (2019). Phase II study of lenvatinib in patients with progressive, recurrent or metastatic adenoid cystic carcinoma. J Clin Oncol.

[32] Di Palma S (2012). Salivary duct carcinomas can be classified into luminal androgen receptor-positive, HER2 and basal-like phenotypes. Histopathology.

[33] Jakob M (2021). Role of cancer stem cell markers ALDH1, BCL11B, BMI-1, and CD44 in the prognosis of advanced HNSCC. Strahlenther Onkol.

[34] Clasen K (2020). PET/MRI and genetic intrapatient heterogeneity in head and neck cancers. Strahlenther Onkol.

[35] Tanadini-Lang S (2020). Radiomic biomarkers for head and neck squamous cell carcinoma. Strahlenther Onkol.

[36] Cozzi L (2019). Predicting survival and local control after radiochemotherapy in locally advanced head and neck cancer by means of computed tomography based radiomics. Strahlenther Onkol.

