# Supplementary material for: High-grade salivary gland cancer: is surgery followed by radiotherapy an adequate treatment to reach tumor control? Results from a tertiary referral centre focussing on incidence and management of distant metastases
Source: Eur Arch Otorhinolaryngol. 2021 Aug 26;279(5):2553–63. doi: 10.1007/s00405-021-07024-9 (PMC8986716; doi:10.1007/s00405-021-07024-9)
Supplement: Supplementary file 1 — Supplementary file1 Univariate analysis of patient-, tumor- and treatment-related parameterst o DFS and DMFS (DOCX 17 KB) [file 405_2021_7024_MOESM1_ESM.docx]

|  | | | **DFS** ρ value Hazard ratio (95% CI) | **DMFS** ρ value Hazard ratio (95% CI) |
| --- | --- | --- | --- | --- |
|  |  |  |  |  |
|  |  |  |  |  |
| **Patient-dependent** | | |  |  |
|  | Tobacco consumption | | 0.850 1.068 (.535 - 2.131) | 0.829 1.087 (.507 - 2.327) |
|  |  | never vs.yes/earlier |  |  |
|  | Sex |  | 0.766 1.101 (.578 - 2.100) | 0.457 07.52 (.351 - 1.609) |
|  |  | male vs. female |  |  |
|  | Age at first diagnosis [y] | | 0.950 1.025 (.466 - 2.255) | 0.889 0.939 (.383 - 2.304) |
|  |  | <70 vs. ≥70 |  |  |
|  | Hypertension | | 0.882 1.054 (.519 - 2.142) | 0.920 1.041 (.475 - 2.279) |
|  |  | no vs. yes |  |  |
| **Tumor-related** | | |  |  |
|  | Primary tumor site | | 0.357 1.358 (.701 - 2.628) | 0.431 1.343 (.639 - 2.824) |
|  |  | Parotid vs. Non-parotid |  |  |
|  | Histologic subtype | | **0.005** 0.393 (.200 - .774) | **0.004** 0.369 (.175 - .780) |
|  |  | adenoid cystic vs. non-adenoid cystic |  |  |
|  | T classification | | **0.009** 2.784 (1.248 - 6.208) | **0.009** 3.321 (1.261 - 8.747) |
|  |  | T1/2 vs. T3/4 |  |  |
|  | primary tumor size [cm] | | 0.859 0.935 (.440 - 1.984) | 0.491 1.315 (.596 - 2.901) |
|  |  | ≤3 vs. >3 |  |  |
|  | N classification | | 0.997 1.001 (.504 - 1.989) | 0.896 1.053 (.481 - 2.304) |
|  |  | N0 vs. N1/2 |  |  |
|  | No. of metastatic lymph nodes | | 0.391 0.918 (.687 - 1.227) | 0.448 0.915 (.665 - 1.258) |
|  |  | ≤1 vs. >1 |  |  |
|  | perinodal spread | | 0.797 0.915 (.462 - 1.814) | 0.626 0.823 (.373 - 1.817) |
|  |  | no vs. yes |  |  |
|  | lymphovascular invasion | | 0.814 1.087 (.537 - 2.202) | 0.475 1.318 (.612 - 2.836) |
|  |  | no vs. yes |  |  |
|  | vascular invasion | | 0.315 1.514 (.664 - 3.451) | 0.227 1.720 (.700 - 4.227) |
|  |  | no vs. yes |  |  |
|  | perineural spread | | 0.359 1.444 (.683 - 3.051) | 0.532 1.331 (.582 - 3.045) |
|  |  | no vs. yes vs. 2 |  |  |
| **Treatment-related** | | |  |  |
|  | Neck dissection | | 0.656 0.870 (.407 - 1.860) | 0.853 1.096 (.481 - 2.498) |
|  |  | no vs. ipsilateral vs. bilateral |  |  |
|  | No. of dissected lymph nodes | | 0.397 0.703 (.306 - 1.613) | 0.420 0.694 (.282 - 1.709) |
|  |  | <10 vs. ≥10 |  |  |
|  |  |  | 0.861 0.938 (.457 - 1.929) | 0.880 1.062 (.482 - 2.337) |
|  |  | ≤27 vs > 27 |  |  |
|  | lymph node density [%] | | 0.938 0.975 (.508 - 1.872) | 0.929 1.034 (.492 - 2.171) |
|  |  | ≤4 vs. >4 |  |  |
|  | resection margins | | 0.128 1.706 (.844 - 3.446) | 0.332 1.484 (.659 - 3.338) |
|  |  | R0 vs. R1/x |  |  |
|  | second primary tumor resection | | 0.802 0.902 (.397 - 2.049) | 0.643 1.219 (.523 - 2.844) |
|  |  | no vs. yes |  |  |
|  | planning target volume | | 0.492 1.386 (.539 - 3.563) | 0.888 1.071 (.409 - 2.804) |
|  |  | primary tumor region vs. Primary tumor region and regional lymph node drainage |  |  |
|  | radiation dose [Gy] | | 0.498 1.426 (.503 - 4.046) | 0.252 2.244 (.534 - 9.432) |
|  |  | <64 vs. ≥64 |  |  |
|  | technique of radiation | | 0.635 0.854 (.441 - 1.653) | 0.897 1.048 (.511 - 2.149) |
|  |  | 3D vs. IMRT and VMAT |  |  |
|  | Radiochemotherapy | | 0.122 1.882 (.825 - 4.293) | 0.075 2.502 (.872 - 7.181) |
|  |  | radiation alone vs. Radiochemotherapy |  |  |

Table S2: Univariate analysis of patient-,tumor- and treatment-related parameterst o DFS and DMFS
