# Supplementary material for: High-grade salivary gland cancer: is surgery followed by radiotherapy an adequate treatment to reach tumor control? Results from a tertiary referral centre focussing on incidence and management of distant metastases
Source: Eur Arch Otorhinolaryngol. 2021 Aug 26;279(5):2553–63. doi: 10.1007/s00405-021-07024-9 (PMC8986716; doi:10.1007/s00405-021-07024-9)
Supplement: Supplementary file 2 — Supplementary file2 Multivariate analysis (DOCX 12 KB) [file 405_2021_7024_MOESM2_ESM.docx]

|  | **DFS** ρ value Hazard ratio (95% CI) | **DMFS** ρ value Hazard ratio (95% CI) |
| --- | --- | --- |
|  |  |  |
|  |  |  |
| Histologic subtype: adenoid cystic vs. non-adenoid cystic | **0.024** 2.497 (1.101 – 5.665) | **0.039** 0.450 (0.211 – 0.961) |
| T classification: T3/4 vs T1/2 | **0.029** 0.456 (0.230 – 0.904) | **0.037** 2.857 (1.068 – 7.646) |

Table S3: Multivariate analysis
